# A Novel Simple Phantom for Verifying the Dose of Radiation Therapy

**DOI:** 10.1155/2015/934387

**Published:** 2015-03-26

**Authors:** J. H. Lee, L. T. Chang, A. C. Shiau, C. W. Chen, Y. J. Liao, W. J. Li, M. S. Lee, S. M. Hsu

**Affiliations:** ^1^Health Physics Division, Institute of Nuclear Energy Research, Longtan 325, Taiwan; ^2^Department of Radiation Oncology, Koo Foundation Sun Yat-Sen Cancer Center, Taipei 112, Taiwan; ^3^Department of Biomedical Imaging and Radiological Sciences, National Yang-Ming University, Taipei 112, Taiwan; ^4^Department of Anesthesiology, China Medical University Hospital, Taichung 404, Taiwan; ^5^Graduate Institute of Clinical Medical Science, China Medical University, Taichung 404, Taiwan; ^6^School of Medical Laboratory Science and Biotechnology, Taipei Medical University, Taipei 110, Taiwan; ^7^School of Medicine, Tzu Chi University, Hualien 970, Taiwan; ^8^Biophotonics and Molecular Imaging Research Center, National Yang-Ming University, Taipei 112, Taiwan

## Abstract

A standard protocol of dosimetric measurements is used by the organizations responsible for verifying that the doses delivered in radiation-therapy institutions are within authorized limits. This study evaluated a self-designed simple auditing phantom for use in verifying the dose of radiation therapy; the phantom design, dose audit system, and clinical tests are described. Thermoluminescent dosimeters (TLDs) were used as postal dosimeters, and mailable phantoms were produced for use in postal audits. Correction factors are important for converting TLD readout values from phantoms into the absorbed dose in water. The phantom scatter correction factor was used to quantify the difference in the scattered dose between a solid water phantom and homemade phantoms; its value ranged from 1.084 to 1.031. The energy-dependence correction factor was used to compare the TLD readout of the unit dose irradiated by audit beam energies with ^60^Co in the solid water phantom; its value was 0.99 to 1.01. The setup-condition factor was used to correct for differences in dose-output calibration conditions. Clinical tests of the device calibrating the dose output revealed that the dose deviation was within 3%. Therefore, our homemade phantoms and dosimetric system can be applied for accurately verifying the doses applied in radiation-therapy institutions.

## 1. Introduction

Cooperation between the International Atomic Energy Agency (IAEA) and the World Health Organization (WHO) led to the implementation of the IAEA/WHO thermoluminescent dosimeter (TLD) postal program for verifying the quality of radiotherapy treatment units. Worldwide TLD-based quality assurance networks confirmed the benefits of this program between 1969 and 1998 [1–4]. In 2007 the IAEA also designed a horizontal arm for use with the originally designed holder for off-axis measurements [5]. In 1997, ESTRO organized a new department, EQUAL, to send TLDs and the IAEA standard holder to participating radiotherapy treatment units in Europe and the Middle East [6]. The organization responsible for radiotherapy quality assurance in the United States is the Radiologic Physics Center (RPC), which is a section of department of the MD Anderson Cancer Center at the University of Texas.

In recent years, the RPC has developed some mailable anthropomorphic dosimetry phantoms for use in different radiotherapy techniques, such as prostate intensity-modulated radiation therapy (IMRT), head and neck IMRT, stereotactic body radiotherapy for the liver and thorax, and stereotaxic radiosurgery [7]. During this time, the number of the participating organizations had increased. The RPC has also provided criteria for acceptable compliance such as the absorbed dose of external beams and brachytherapy and the mechanical parameters of linear accelerators (LINACs). The most important criterion has been for the delivered tumor dose to be within 5% of the target dose.

Multipurpose phantoms have also been designed in several countries (e.g., the Czech Republic and Poland) for measuring multiple parameters simultaneously [8–14]. The aim of the present study was to design a lightweight, low-workload, easy-to-set-up, and transportable phantom for use in mailed TLD postal audit programs. This was achieved by considering dosimeter selection, dose-response calibration, phantom scatter correction, and energy-dependent correction.

## 2. Material and Methods

The primary experiment of this study involved verifying the dose outputs of the photon and electron beams produced by medical LINACs. The cubic TLDs (TLD-100) were used in this study. A ^60^Co source established at the Institute of Nuclear Energy Research (INER, Taiwan) was used to irradiate the dosimeters. The dosimeter remote-monitoring system was tested at the INER for accuracy, stability, and energy dependence.

### 2.1. Phantom Design

Various dosimeters or phantoms have been developed for postal audit programs in recent decades. Such mailable audit systems have been designed to be inexpensive, lightweight, and easy to set up precisely. Nonetheless, these phantoms also have some disadvantages, such as the inconvenience of a water tank being involved with the IAEA holder and the sizes and measuring depths of RPC phantoms change with the beam modality and beam energy.

**Figure 1:**
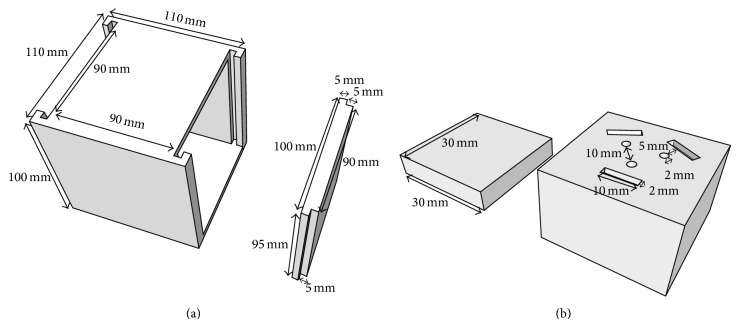
(a) Self-designed simple phantom. (b) Dosimeter hole in homemade phantoms.

With the aim of improving the efficiency and accuracy of mailable phantoms, we designed an acrylic phantom with features of smallness, simple geometry, and depth adjustment. The dimensions of this phantom are 110 × 110 × 100 mm^3^ (Figure 1(a)), with its internal space containing three 90 × 90 × 30 mm^3^cubic layers. One layer contains a hollowed-out 30 × 30 × 30 mm^3^ hole to allow the TLD columns to be changed. The TLD columns are divided into two parts. For the direction of irradiation, the thickness of the upper part depends on the measuring depth; the other part contains three circular and three rectangular holes in which dosimeters can be placed, as shown in Figure 1(b). In order to increase the accuracy of the experiment, we can obtain the six TLD readings in a single irradiation. Although some aspects such as a wide field measurement range and depth variation are not compatible with small acrylic phantoms, our phantom offers a solution to the depth limitation by providing the ability to adjust the three layers in the different direction.

### 2.2. TLD Calibration Procedures

When developing a dose-verification system, we needed to know which TLDs are sufficiently stable to use as postal dosimeters. There are various types of TLD available, which makes it difficult to ensure linearity between responses and doses. As a first step, we used a TLD reader (Harshaw 5500) to identify which TLDs in the same batch were unstable. By delivering doses of 80, 160, 240, and 80 cGy using the ^60^Co source at the INER, we compared the batch average response with the response of each TLD for the same dose.

A dose-response curve is needed to convert the TLD response into the absorbed dose after irradiation. We divided these stable TLDs (coefficient of variance < 3%) into 10 dose calibration points: 10, 20, 30, 50, 100, 200, 300, 400, 500, and 800 cGy. After analyzing the TLD readout values, we used the determination coefficient (*R* ^2^) of the linear fitting curve to assess the linearity of the dose-response curve. An *R* ^2^ value greater than 0.99 was considered to indicate that the formula was sufficiently accurate for converting the TLD response into the actual radiation dose.

### 2.3. Determination of Absorbed Dose in Water

A TLD with a response uncertainty of within 3% was used in this study. The absorbed dose in water for high-energy photon and electron beams was obtained using the following equation:

(1) DoseTLD,Co60=R×CF×fe×fp×fs,

where

 Dose is the absorbed dose in water for high-energy beams,
 
*R* is the average response of the TLD,
 CF is the dose-response conversion factor,
 
*f*_*e*_ is the energy-dependence correction factor,
 
*f*_*p*_ is the phantom scatter correction factor,
 
*f*_*s*_ is the setup-condition factor.

#### 2.3.1. Energy-Dependence Correction Factor (*f*_*e*_)

In verifying the dose of radiation therapy, the main objective was to confirm the radiation doses commonly used in radiotherapy units, such as 6 and 10 MV photons and 6, 9, and 12-MeV electron beams. Because the TLD responses differ somewhat between high-energy beams and ^60^Co, the energy-dependence correction factor (*f*_*e*_) should be utilized in determinations of the absorbed dose in water. To obtain *f*_*e*_ for each phantom, we first used an ion chamber (Wellhofer FC65-P) in accordance with the AAPM TG-21 protocol to calibrate the beam output of the Varian LINAC using the air-kerma calibration factor (*N*_*k*_ = 4.4482 × 10^7^ Gy/C) provided in the INER ion-chamber dose calibration report. After irradiating the TLDs with a known dose using high-energy beams and ^60^Co, the response to the unit dose represented the sensitivity, and this value was normalized to that for ^60^Co as the energy-dependence correction factor according to

(2) fe=TLD  response  of  unit  dose  from  Co-60 source ×TLD  response  of  unit  dose     from  high-energy  beams−1.

#### 2.3.2. Phantom Scatter Correction Factor (*f*_*p*_)

The size of a phantom influences the scattering dose and the charged-particle equilibrium. Correcting for the scattering dose between these phantoms and a water phantom is necessary. Under the condition of fixed distances between the source and the chamber for the photon and electron beams, we used the ion chamber to measure the ionization ratio of these small acrylic phantoms and the full-scatter solid water (30 × 30 × 30 cm^3^) for high-energy beams: 6 and 10 MV photon beams and 6, 9, and 12 MeV electron beams. According to AAPM TG-21, the absorbed dose in medium is defined as

(3) $$\begin{array}{c} D_{\text{med}}\left(d_{\max}\right)=M_{\text{corrected}}N_{\text{gas}}P_{\text{repl}}P_{\text{wall}}{\left(\frac{\overset{-}{L}}{\rho }\right)}_{\text{gas}}^{\text{med}}. \end{array}$$

Because the same ionization chamber was used to measure the *D*_med_ values of acrylic and water, the *N*_gas_, *P*_repl_, and *P*_wall_ parameters are the same in these two mediums. Therefore, the dose ratio for the solid water and postal phantoms was defined as the phantom scatter correction factor, calculated using the ionization ratio and the ratio of the mean restricted mass collision stopping power:

(4) phantom  scatter  correction  factor =DwaterDarcylic=MwaterMarcylicL−ρarcylicwater.

Considering the phantom stability, we also used three different types of LINACs (produced by Varian, Siemens, and Elekta) and irradiated the measuring point of each phantom at 100 MU as calculated in the reference condition.

#### 2.3.3. Setup-Condition Factor (*f*_*s*_)

The dose calibration conditions differ for each LINAC when using photon and electron beams. These following reference points are commonly used: (1) photon beams-SSD = 100 cm, depth = *d*_max⁡_ or SAD = 100 cm, depth = *d*_max⁡_ or SSD = 100 cm, depth = 5 cm; and (2) electron beams-SSD = 100 cm, depth = *d*_max⁡_.

Therefore, the factor used to convert the dose in a mailable phantom varies with the dose calibration conditions at each hospital. The setup-condition factors calculated for the beam data included the tissue-air ratio (TAR), tissue-phantom ratio (TPR), percentage depth dose, and inverse-square law.

**Figure 2:**
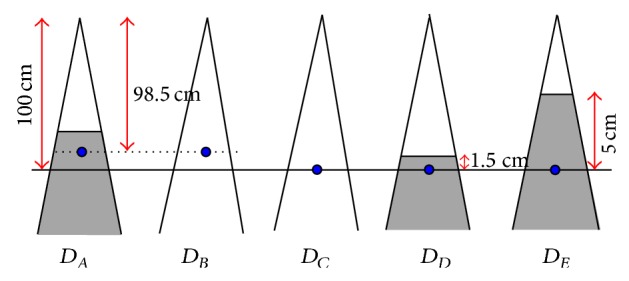
The schematic of setup condition.

As an example, for 6 MV photon beams, the dose reference point is represented as *D*_*E*_ (SAD = 100 cm, depth = 5 cm), which means that 1 MU is equal to 1 cGy. Referring to (5), after applying the phantom scatter correction factor in (1), the dose at a particular depth in water is *D*_*A*_. Then, the TAR and inverse-square law could be used to convert *D*_*A*_ into *D*_*B*_, *D*_*C*_, and *D*_*D*_. Finally, according to the clinical units providing TPR value, the *D*_*D*_ was converted to *D*_*E*_ (shown in Figure 2):

(5) $$\begin{array}{c} \frac{D_{B}}{D_{A}}=\frac{1}{\text{TAR}\left(d=1.5\;\text{cm},\;\text{field}\;\text{size}=9.85\times 9.85\;\text{c}{\text{m}}^{2}\right)},\\ \frac{D_{C}}{D_{B}}={\left(\frac{98.5}{100}\right)}^{2},\\ \frac{D_{D}}{D_{C}}=\text{TAR}\left(d=1.5\;\text{cm},\;\text{field}\;\text{size}=10\times 10\;{\text{cm}}^{2}\right),\\ \frac{D_{E}}{D_{D}}=\frac{1}{\text{TPR}\left(d=1.5\;\text{cm},\;\text{field}\;\text{size}=10\times 10\;{\text{cm}}^{2}\right)}. \end{array}$$

## 3. Results

### 3.1. TLD Calibration

**Figure 3:**
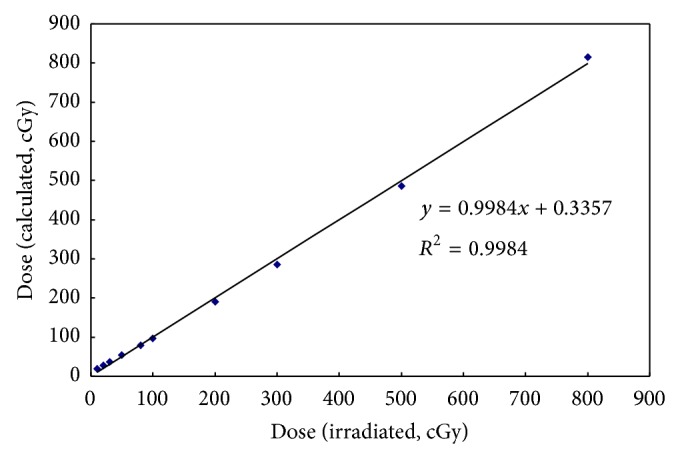
The dose linearity of TLD (calculated dose versus irradiated dose).

The above-described dose calibration procedures allowed the TLD response to be analyzed for 10 dose points. The linear trend line equation was used to convert the TLD response to the radiation dose. After checking the feasibility, as indicated by *R* ^2^ exceeding 0.99, the irradiated and calculated doses for the TLDs were confirmed as being linearly dependent (Figure 3).

### 3.2. Energy-Dependence Correction Factor

**Figure 4:**
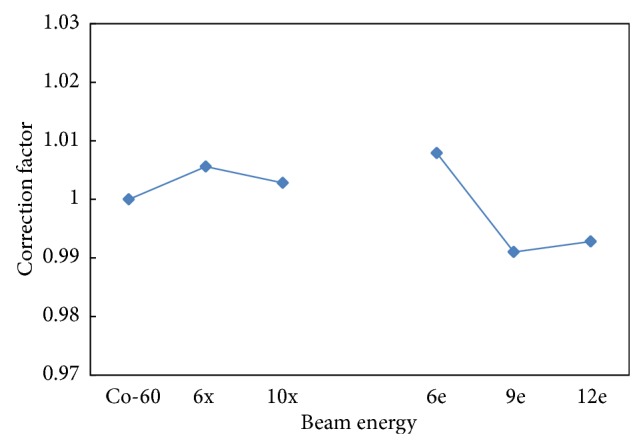
The energy-dependence correction factor of TLD.

The energy dependence of high-energy beams compared with the ^60^Co source at the INER is shown in Figure 4. The *f*_*e*_ values were 1.005 and 1.003 for the 6 and 10-MV photon beams, respectively, and 1.006, 0.991, and 0.993 for the 6, 9, and 12-MeV electron beams. The standard deviation of *f*_*e*_ was 0.73%, with no apparent variance.

### 3.3. Phantom Scatter Correction Factor

**Figure 5:**
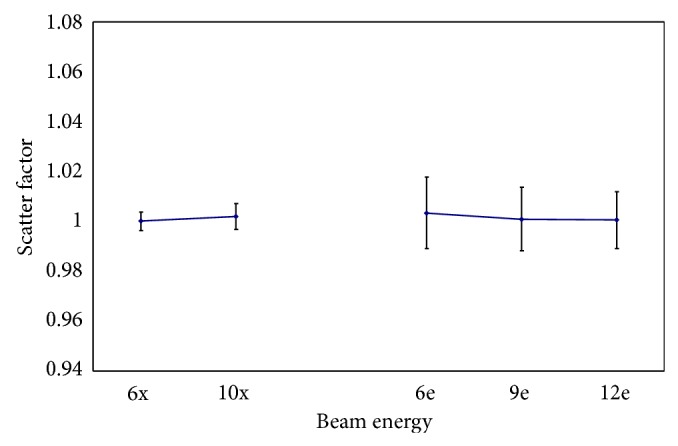
Scatter correction factor of phantom.

The phantom scatter correction factors of the homemade self-designed phantoms for each beam energy produced by the five LINACs are shown in Figure 5. For the homemade phantoms, this factor was stable and near to 1 for each of the high-energy beams. The results for the energy-dependence correction factors indicated that the properties of the TLD-100 were stable. This reflects the small variance in the interaction with high-energy beams due to the lower atomic number.

In order to check the validity of the audit procedures and the relative parameters, we used the ion chamber and followed AAPM TG-21 to measure the daily output dose. TLDs combined with two types of phantoms were exposed to 200 MU. Applying (1), the TLD response was converted to the absorbed dose in water with full scatter. The doses measured by the ionization chamber and TLDs are compared in Table 1. Most of the differences between the beams in the phantoms are within ±3%, which confirms the accuracy and stability of the phantom designed in this study.

## 4. Conclusion

Radiation-therapy institutions worldwide participate in postal audit programs. This study investigated this process based on consideration of the procedures of phantom setup, dose delivery, and the formula for the absorbed dose in water. The derived equations facilitated the easy comparison of the reproducibility and accuracy of TLD systems. The stable TLDs were then used in a dose-verification system, and the dose calibration curve was found to be suitable for converting the TLD readout value into an accurate dose. After comparing with the measurements made using an ionization chamber and following AAPM TG-21, the analogous doses calculated from TLDs indicated the validity of the correction factors in (1). The light weight, full scattering dose, and depth-adjusting features of the designed phantoms allow them to be used to verify other medical-physics parameters such as the beam quality, output factors, and wedge factors.

Powder TLDs used by the IAEA in postal audit programs have some advantages, such as being waterproof and highly accurate, but the present study has demonstrated that cubic TLDs provide more convenient measurements with a low workload. The designed phantoms and operating procedures reported herein can be used to verify the doses applied in radiation-therapy institutions.

## Figures and Tables

**Table 1:** The dose comparison between ionization chamber and TLDs.

	*D*_TLD_ (cGy)	*D*_IC_ (cGy)	Difference (%)
6 MV photon	194.48 ± 2.32	199.50 ± 1.92	−2.519%
10 MV photon	197.21 ± 3.93	197.97 ± 1.87	−0.386%
6 MeV electron	203.71 ± 2.04	199.32 ± 1.56	2.205%
9 MeV electron	194.84 ± 3.62	198.45 ± 2.03	−1.819%
12 MeV electron	198.99 ± 3.13	198.04 ± 1.58	0.478%

*D*_TLD_: dose measured from TLDs; *D*_IC_: dose measured from the ionization chamber.
